# Silent Risks in the Blood Supply: Co‐Inherited Glucose‐6‐Phosphate Dehydrogenase Deficiency and Sickle Cell Trait Among Donors at the Cape Coast Teaching Hospital, Ghana: A Cross‐Sectional Study

**DOI:** 10.1002/hsr2.72785

**Published:** 2026-07-07

**Authors:** Bashirudeen Kofi Mensa Essel, Walter Quarm, Comfort Fynn, Rebecca Peniel Storph, Evans Duah, Edward Morkporkpor Adela, Emmanuel Ofori, Nana Benyin Aidoo, Patrick Adu

**Affiliations:** ^1^ Laboratory Department Cape Coast Teaching Hospital Cape Coast Ghana; ^2^ Department of Medical Laboratory Science, School of Allied Health Sciences, College of Health and Allied Sciences University of Cape Coast Cape Coast Ghana; ^3^ Urology Department Cape Coast Teaching Hospital Cape Coast Ghana; ^4^ Research Unit Cape Coast Teaching Hospital Cape Coast Ghana

**Keywords:** co‐inheritance of G6PD deficiency and SCT, G6PD deficiency, prevalence, SCT (sickle cell hemoglobin variants), sickle cell traits

## Abstract

**Background and Aims:**

Blood transfusion saves lives, but red cell enzymopathies and hemoglobin variants can reduce red cell survival under oxidative stress. In sub‐Saharan Africa, blood donors are not routinely screened for glucose‐6‐phosphate dehydrogenase (G6PD) deficiency or sickle cell trait (SCT). Evidence on co‐inheritance in donors is limited, despite possible harm to vulnerable recipients such as neonates and chronically transfused patients. This study determined the prevalence of G6PD deficiency, SCT, and their co‐inheritance among blood donors, and assessed donor awareness and selected laboratory findings relevant to transfusion safety.

**Methods:**

A hospital‐based cross‐sectional study was conducted between February and August 2023 among 250 blood donors aged 16–50 years selected through simple random sampling. Following informed consent, participants completed a structured questionnaire. G6PD status was determined using the methemoglobin reduction test, while sickling was screened using sodium metabisulfite. Hemoglobin electrophoresis was used to determine hemoglobin phenotype, and Full Blood Count (FBC) analysis was performed using the Sysmex XN‐350 hematology analyzer. Data were analyzed using SPSS version 26.0, with *p* < 0.05 considered statistically significant.

**Results:**

The mean age was 21.8 ± 6.3 years. Most donors were males [146/250 (58.4%)], students [184/250 (73.6%)], and first‐time donors (174/250, 69.6%). Most donors did not know their G6PD status (246/250, 98.4%), blood group (167/250, 66.8%), or sickling status (235/250, 94%). Laboratory testing showed 35/250, 14% had full G6PD deficiency, 55/250, 22% had sickle cell trait (predominantly Hb AS), and 15/250, 6% co‐inherited both conditions. Blood group O Rh “D” positive was the most common (143/250, 57.2%). G6PD status and full blood count parameters differed by sex (*p* < 0.05).

**Conclusion:**

The 6% co‐inheritance of G6PD deficiency and SCT among blood donors, together with low donor awareness, highlights potential transfusion safety concerns. Targeted donor education, pre‐donation counseling, and screening policies may improve transfusion safety in high‐prevalence settings.

## 1 Introduction

Blood transfusion is an essential therapeutic procedure that saves patients with severe anemia [1, 2]. It remains the comprehensive management or treatment for patients with sickle cell disease (SCD), as they may require transfusion for indications specific to their disease. Also, transfusions are done to correct bleeding disorders and improve immunity [3]. Annually, there is an estimation that over 118.5 million blood units are collected on a global scale [4]. The rate of collection across nations indicates that the blood donation rate in high‐income countries is 33.1 donations per 1000 people; 11.7 donations in middle‐income countries, and 4.6 donations in low‐income countries [4].

Blood donors are screened for transfusion transmissible infections (TTIs): Hepatitis B virus (HBV), Hepatitis C virus (HCV), Syphilis, Human Immunodeficiency Virus I and II (HIV I and II) [4]. The World Health Organization (WHO) recommends screening of all blood donations before use. However, a few (16) countries are unable to screen for all the underlisted TTIs, attributed to the unavailability of test kits or appropriate equipment for screening [4]. Thus, the prevalence of TTIs in blood donation is high in low‐income countries compared to high‐income countries [4]. Aside from the TTIs to be screened before transfusion, several current research findings point out a red cell metabolism defect and hemoglobinopathy to be considered in blood donation.

Hemoglobinopathy is a blood disorder caused by both abnormal hemoglobin (Hb) structure production (e.g., HbS and HbC) and reduced globin synthesis (e.g., thalassemia). Whereas glucose‐6‐phosphate dehydrogenase (G6PD) deficiency is caused by a red cell metabolism defect [5, 6]. SCD and G6PD enzymopathy are both recessively inherited. The sickle cell gene is autosomally inherited, whereas G6PD is sex‐linked. SCD occurs due to the substitution of valine for glutamic acid in position 6 in the beta globin [7, 8, 9, 10]. This mutation leads to the production of abnormal hemoglobin (Hb S) instead of normal hemoglobin (Hb A) [7, 10, 11, 12]. Sickle cell trait (Hb AS) occurs as a result of heterozygous inheritance, whereas SCD occurs as a result of homozygous inheritance [5, 11]. G6PD deficiency is an X‐linked enzymopathy which is caused by mutations in the G6PD gene [13, 14, 15]. It has approximately 230 different causative mutations for this enzyme deficiency [16]. Some mutations are specific to the location or racial group, with the most common being the A variant and the Mediterranean variant [17]. The effects of G6PD deficiency are expressed more in males than in females [13]. It is a key enzyme in the pentose phosphate pathway (PPP), which helps to prevent oxidative damage in red cells [12, 13, 14, 18, 19]. There is an estimation of about 400 million people being G6PD deficient worldwide [5, 6, 13].

SCT and G6PD deficiency are inherited independently. During blood donation, donors are interviewed and screened for TTIs before donation, and their blood product is used for transfusion. However, less emphasis is placed on the donor's SCT and G6PD status, as several studies have shown adverse effects of SCT and G6PD‐deficient blood on the recipients of such blood [20]. A review study conducted by Renzaho et al. found that most of the studies involved in the review reported that G6PD deficient blood transfused to children had a more deleterious effect than in adults. It was revealed that total serum bilirubin was abnormally increased in an infant transfused with G6PD‐deficient blood from 6 hours up to 60 hours after transfusion, then recommended routine screening of G6PD deficiency before transfusion [20]. Another study identified that the quality of blood donated from SCT and G6PD‐deficient donors might be altered during processing, storage or in the recipients' circulatory system [21]. Other studies pointed out the high possibility for their co‐inheritance (SCT and G6PD) as these pathologies have been shown to protect against malaria infection, especially in sub‐Saharan Africa [10]. Of the numerous studies conducted on blood donation and transfusion, especially in Africa, blood donors are not routinely screened for SCT and G6PD. The effects and risks of using SCT and G6PD‐deficient blood during transfusion are not established properly as well. There is a high possibility of using SCT and G6PD blood unknowingly for transfusion. This study sought to assess the prevalence of G6PD deficiency and SCT among blood donors at the Cape Coast Teaching Hospital, Ghana.

## 2 Methodology

### 2.1 Study Site

This study was conducted at the Cape Coast Teaching Hospital's Transfusion Medicine Unit in the Central Region of Ghana. Cape Coast Teaching Hospital (CCTH) is a tertiary health facility and has a bed capacity of 400. CCTH serves as a referral center for the Central Region and parts of Western, Western North, and Ashanti Regions.

### 2.2 Study Design

This study was a hospital‐based cross‐sectional study. A simple random sampling technique was used in participant selection. This study was conducted between February and August 2023. The study recruited donors between the ages of 16 and 50 years who tested negative for the TTIs. This study excluded individuals who had been transfused with blood for the past 3 months. Also, donors who were on medications (such as cotrimoxazole, aspirin, fansidar, and nitrofurantoin) known to affect G6PD enzyme activity were excluded [22]. Participants read and signed written informed consent before being enrolled. Participants were also informed of their withdrawal rights from the study at any point in time and assured that their medical records would be kept and treated with strict confidentiality.

### 2.3 Sample Size/Population

The prevalence of SCT and G6PD enzymopathy coinheritance among blood donors at Berekum was 7% [1]. The prevalence rate from that study was used to calculate the sample size to be 100. A final sample size of 250 study participants was then chosen to account for nonresponse, illustrated as follows.

The sample size for the study was calculated using Fisher's sampling formula:

$$N=Z^{2}P\left(1-P\right)/D^{2}$$

where *N* represents the estimated sample size, *Z* represents the constant for 95% confidence interval given as 1.96, *P* represents the average prevalence of SCT and G6PD enzymopathy coinheritance, which was 7% obtained from a study conducted among blood donors in Berekum of the Bono Region, and *D* represents the percentage margin of error, taken as 5%. Therefore, the study participants consisted of 250 prospective blood donors at the Cape Coast Teaching Hospital's Transfusion Medicine Unit.

### 2.4 Statistical Analysis

Data was entered into a Microsoft Excel spreadsheet and subsequently exported to IBM SPSS Statistics (Version 26.0) for analysis. Descriptive statistics were computed to summarize donor characteristics, awareness levels, and laboratory findings. The normality of continuous variables was assessed using the Shapiro–Wilk test (*p *> 0.05). Categorical variables were presented as frequencies (*n*) and percentages (%), whereas continuous variables were expressed as mean ± standard deviation (SD) or mean ± standard error (SE) with 95% confidence intervals (CI) for normally distributed data. The *χ* ^2^ test was used to examine differences in categorical laboratory variables, including blood group, glucose‐6‐phosphate dehydrogenase (G6PD) status, and hemoglobin electrophoresis, between male and female donors. Differences in continuous hematological parameters between males and females were assessed using the independent samples *t*‐test. Statistical significance was determined using two‐tailed *p* values, with *p* < 0.05 considered statistically significant. Results were presented in tables.

### 2.5 Ethical and Institutional Approvals

All protocols for the study were approved by the Cape Coast Teaching Hospital Ethical Review Committee (Ethical Clearance REF: CCTHERC/EC/2023/076). Also, approval was sought from the head of the Cape Coast Teaching Hospital's Transfusion Medicine Unit.

### 2.6 Questionnaire Administration

A structured, pre‐tested questionnaire seeking information such as social data (e.g., age, sex, etc.), knowledge about their status (e.g., G6PD and sickling), transfusion history and type of donor (e.g. commercial, replacement, etc.) was used. Before providing the information, participants were taken through a process of filling out the questionnaire through an interview format. Participants who were unable to write were assisted in getting reliable information.

### 2.7 Sample and Collection

Four milliliters of whole blood samples were taken from the participants using a 5cc syringe into an Ethylene diamine tetra acetic acid (EDTA) anticoagulated tube for the Qualitative G6PD test, Sickling test, Blood group, Full Blood Count (FBC), and Hb electrophoresis.

### 2.8 Laboratory Investigations

Blood samples were worked on immediately after they were taken. Qualitative G6PD, sickling, FBC, blood group and hemoglobin electrophoresis tests were performed using the standard operating procedures as per previously published protocols [23, 24].

#### 2.8.1 Methemoglobin Reduction Test (G6PD Screening)

G6PD screening was conducted via the methemoglobin reduction assay, as detailed by Cheesbrough [23]. G6PD status was reported as partial defect (PD), full defect (FD), or no defect (ND).

The methemoglobin reduction test was selected for screening G6PD deficiency because it is a cost‐effective, rapid, and widely used qualitative assay suitable for resource‐limited settings. The test is based on the ability of red cells with normal G6PD activity to reduce methemoglobin to hemoglobin via NADPH‐dependent pathways. It has moderate sensitivity (approximately 85%–95%) for detecting severe deficiency, but reduced sensitivity in heterozygous females due to lyonization. Its advantages include simplicity and low cost. However, it cannot reliably quantify enzyme activity or detect mild/intermediate deficiency [23].

#### 2.8.2 Sickling Test (Sodium Metabisulphite Method)

The sickling test was done as per a previously published protocol [23]. The sickling status was reported as negative and positive.

The sodium metabisulfite (sickling) test was used for initial screening of sickle cell status because it induces hypoxic conditions that promote HbS polymerization, resulting in red cell sickling. This method is simple, inexpensive, and suitable for large‐scale screening. However, it has limited specificity and cannot differentiate between sickle cell trait (HbAS) and sickle cell disease (HbSS), necessitating confirmatory testing such as hemoglobin electrophoresis. Its sensitivity is generally high (> 90%) for detecting HbS, but it may miss low‐concentration variants [23].

#### 2.8.3 Hemoglobin Electrophoresis Test

Cellulose acetate electrophoresis (pH 8.2–8.6) was employed to identify hemoglobin variants in the participants, following established protocols [23]. The Hb electrophoresis was reported as Hb A, Hb AC, Hb AS, and Hb C.

#### 2.8.4 Blood Group Test

The status of participants' blood group was achieved in accordance with established protocols [23]. The blood group was reported as O Rh “D” Positive, O Rh “D” Negative, A Rh “D” Positive, A Rh “D” Negative, B Rh “D” Negative, B Rh “D” Positive, AB Rh “D” Positive, and AB Rh “D” Negative.

#### 2.8.5 Full Blood Count

Using the manufacturer's protocol [24], the Sysmex XN‐350 hematology analyzer (Sysmex Corporation, Kobe, Japan) was employed to determine each participant's FBC.

## 3 Results

Table 1 describes the demographic characteristics of the donor participants. The mean age of donors was 21.8 ± 6.26 years; most were males (146/250, 58.4%), had attained senior high school education (203/250, 81.2%), were single (221/250, 88.4%), and lived in urban areas (126/250, 50.4%). Most participants were students (184/250, 73.6%) and voluntary donors (185/250, 74.0%), with the majority being first‐time donors (174/250, 69.6%). Among donations for which facility data were available (*n* = 79), most took place at Cape Coast Teaching Hospital (64/79, 81.0%).

**Table 1:** Donors' demographic information.

Variable	*N* = 250, *n* (%)
Mean age ± SD	21.8 ± 6.26
Sex	
Male	146 (58.4)
Female	104 (41.6)
Highest educational level	
No school	4 (1.6)
Junior high school	16 (6.4)
Senior high school	203 (81.2)
Tertiary	27 (10.8)
Residence	
Rural	124 (49.6)
Urban	126 (50.4)
Marital status	
Single	221 (88.4)
Married	29 (11.6)
Occupation	
Unemployed	3 (1.2)
Student	184 (73.6)
Civil servant	24 (9.6)
Self‐employed/artisan	38 (15.2)
Professional	1 (0.4)
Type of donor	
Voluntary	185 (74.0)
Replacement	65 (26.0)
First‐time donor?	
No	76 (30.4)
Yes	174 (69.6)
Number of times donated	
Once	35 (46.7)
Twice	20 (26.7)
Thrice	7 (9.3)
More than thrice	8 (10.7)
Regular donor	5 (6.6)
Donation facility (*n* = 79)	
Ajumako polyclinic	1 (1.3)
Biriwa NVTI	4 (5.0)
Bonn clinic	1 (1.3)
CCTH	64 (81.0)
CCMH	1 (1.3)
Ewim	1 (1.3)
Adisadel college	1 (1.3)
CCTU	1 (1.3)
St. Francis Xavier hospital	1 (1.3)
Trauma and specialist hospital	1 (1.3)
Twifo Praso government hospital	3 (3.7)

Donors' awareness of their clinical history and general knowledge of blood transfusion is exhibited in Table 2. Most participants were unaware of TTIs (179/250, 71.6%). Among those who were aware, HIV I and II were the most known infection (67/172, 38.9%), followed by syphilis (37/172, 21.6%) and HBV (32/172, 18.6%), while HCV was the least known (16/172, 9.3%). Only 2/250 donors (0.8%) reported known chronic medical conditions, liver disease (1/2, 50.0%), and ulcer (1/2, 50.0%), both of whom were medicated, had been admitted for the condition, and did not require a blood transfusion. Most donors had no travel history outside Ghana (242/250, 96.8%). The majority of participants were unaware of their G6PD status (246/250, 98.4%), blood group type (167/250, 66.8%), and sickling status (235/250, 94.0%).

**Table 2:** Donor's awareness of clinical history and general knowledge of blood transfusion.

Variable	*N* = 250, *n* (%)
Are you aware of any form of viral infection or illness transmitted through blood transfusion?	
No	179 (71.6)
Yes	71 (28.4)
Donor's perceived diseases associated with blood transfusion (multiple responses) (*n* = 172)	
Gonorrhea	20 (11.6)
HIV I and II	67 (38.9)
HBV	32 (18.6)
HCV	16 (9.3)
Syphilis	37 (21.6)
Any known chronic medical condition?	
No	248 (99.2)
Yes	2 (0.8)
Donor's clinical status (*n* = 2)	
Liver disease	1 (50.0)
Ulcer	1 (50.0)
Medication for medical condition (*n* = 2)	
Inhaler	1 (50.0)
Omeprazole	1 (50.0)
Have you been admitted on account of this condition before? (*n* = 2)	
No	0 (0.0)
Yes	2 (100.0)
Did you require blood transfusion? (*n* = 2)	
No	2 (100.0)
Yes	0 (0.0)
Do you have a travel history outside Ghana?	
No	242 (96.8)
Yes	8 (3.2)
Country of visit (*n* = 8)	
Benin	1 (12.5)
Ivory Coast	1 (12.5)
Nigeria	2 (25.0)
Togo	4 (50.0)
Year of travel (*n* = 8)	
2009	2 (25.0)
2014	1 (12.5)
2016	1 (12.5)
2017	1 (12.5)
2021	1 (12.5)
2022	2 (25.0)
Do you know your G6PD status?	
No	246 (98.4)
Yes	4 (1.6)
Do you know your blood group?	
No	167 (66.8)
Yes	83 (33.2)
Do you know your Sickle Cell status?	
No	235 (94.0)
Yes	15 (6.0)

Table 3 presents the distribution of blood group type, G6PD status, and sickle cell trait (SCT) among blood donors and differences between males and females. O Rh “D” positive was the most common blood group (143/250, 57.2%), followed by B Rh “D” positive (41/250, 16.4%). The majority of donors were normal for G6PD (ND, 213/250, 85.2%), sickling negative (220/250, 88.0%), and had normal hemoglobin type (Hb A, 195/250, 78.0%). A smaller proportion carried sickle cell traits (Hb AS, 30/250, 12.0%). Fifteen donors (15/250, 6.0%) co‐inherited G6PD deficiency and SCT, of whom 14/15 (93.3%) had full G6PD deficiency with SCT and 1/15 (6.7%) had partial G6PD deficiency with SCT. Among these variables, only G6PD status differed significantly by sex, *χ* ^2^ (2, *N* = 250) = 14.93, *p* = 0.001, with fully deficient G6PD observed in 30/35 (85.7%) males compared to 5/35 (14.3%) females.

**Table 3:** Laboratory investigation on donor blood (blood group, G6PD, and Hb electrophoresis) stratified by sex.

Variable	Total *N* = 250 *n* (%)	Male *N* = 146 *n* (%)	Female *N* = 104 *n* (%)	*χ* ^2^	*p* value
Blood group				1.37	0.965
O Rh “D” positive	143 (57.2)	84 (58.7)	59 (41.3)		
O Rh “D” negative	13 (5.2)	9 (69.2)	4 (30.8)		
A Rh “D” positive	39 (15.6)	21 (53.9)	18 (46.1)		
A Rh “D” negative	4 (1.6)	2 (50.0)	2 (50.0)		
B Rh “D” positive	41 (16.4)	24 (58.5)	17 (41.5)		
B Rh “D” negative	4 (1.6)	2 (50.0)	2 (50.0)		
AB Rh “D” positive	6 (2.4)	4 (66.7)	2 (33.3)		
G6PD				14.93	0.001*
ND	213 (85.2)	116 (54.5)	97 (45.5)		
PD	2 (0.8)	0 (0.0)	2 (100.0)		
FD	35 (14.0)	30 (85.7)	5 (14.3)		
Sickling				0.04	0.837
Negative	220 (88.0)	129 (58.6)	91 (41.4)		
Positive	30 (12.0)	17 (56.7)	13 (43.3)		
HB Electrophoresis				6.02	0.083
Hb A	195 (78.0)	110 (56.4)	85 (43.6)		
Hb AC	24 (9.6)	19 (79.2)	5 (20.8)		
Hb AS	30 (12.0)	17 (56.7)	13 (43.3)		
Hb C	1 (0.4)	0 (0.0)	1 (100.0)		
Sickle cell trait (SCT)				1.44	0.229
SCT	55 (22.0)	36 (65.5)	19 (34.5)		
No SCT	195 (78.0)	110 (56.4)	85 (43.6)		
Co‐inheritance (*n* = 15)				0.07	1.000
G6PD FD + SCT	14 (93.3)	13 (92.9)	1 (7.1)		
G6PD PD + SCT	1 (6.7)	0 (0.0)	1 (100.0)		

* Significance exists at *p* value < 0.05.

Laboratory investigations of donor blood are presented in Table 4. Among the full blood count parameters assessed, significant differences in mean values between males and females were observed for white blood cells (WBC, *p *= 0.033), red blood cells (RBC, *p* < 0.001), hemoglobin (Hb, *p *< 0.001), hematocrit (HCT, *p *< 0.001), mean cell volume (MCV, *p *< 0.001), platelets (PLT, *p *= 0.012), and monocytes (MONO, *p *= 0.041).

**Table 4:** Laboratory investigation on donor blood (full blood count) stratified by sex.

Variable	Combined mean ± SE (CI)	Male mean ± SE (CI)	Female mean ± SE (CI)	*t*‐statistic	*p* value
WBC	6.9 ± 0.21 (6.5–7.4)	6.6 ± 0.27 (6.1–7.1)	7.5 ± 0.32 (6.8–8.1)	2.14	0.033*
RBC	4.6 ± 0.03 (4.6–4.7)	4.7 ± 0.04 (4.7–4.8)	4.5 ± 0.04 (4.4–4.5)	−5.24	< 0.001*
HB	13.5 ± 0.07 (13.4–13.6)	13.8 ± 0.09 (13.6–14.0)	13.0 ± 0.09 (12.9–13.2)	−5.91	< 0.001*
HCT	39.3 ± 0.20 (38.9–39.7)	40.2 ± 0.27 (39.7–40.8)	37.9 ± 0.26 (37.5–38.5)	−5.84	< 0.001*
MCV	81.9 ± 0.40 (81.1–82.7)	82.6 ± 0.51 (81.6–83.6)	80.9 ± 0.64 (79.6–82.1)	−2.19	< 0.001*
MCH	28.2 ± 0.19 (27.8–28.6)	28.4 ± 0.23 (27.9–28.9)	27.8 ± 0.34 (27.2–28.5)	−1.49	0.137
MCHC	34.1 ± 0.12 (33.8–34.3)	34.2 ± 0.15 (33.9–34.5)	33.9 ± 0.18 (33.5–34.2)	−1.24	0.216
PLT	206.9 ± 4.73 (197.6–216.2)	196.9 ± 6.03 (184.9–208.8)	221.0 ± 7.41 (206.3–235.7)	2.54	0.012*
NEUT	4.10 ± 0.19 (3.71–4.49)	3.84 ± 0.26 (3.33–4.35)	4.47 ± 0.31 (3.86–5.07)	1.56	0.121
LYMPH	2.11 ± 0.07 (1.97–2.24)	2.03 ± 0.09 (1.85–2.22)	2.22 ± 0.10 (2.02–2.42)	1.33	0.186
MONO	0.58 ± 0.02 (0.55–0.62)	0.55 ± 0.03 (0.50–0.59)	0.63 ± 0.03 (0.57–0.69)	2.06	0.041*
EO	0.12 ± 0.01 (0.10–0.14)	0.12 ± 0.01 (0.10–0.15)	0.12 ± 0.02 (0.1–0.2)	0.03	0.976
BASO	0.03 ± 0.00 (0.03–0.04)	0.03 ± 0.00 (0.03–0.04)	0.03 ± 0.00 (0.03–0.04)	−0.47	0.637

* Significance exists at *p* value < 0.05.

## 4 Discussion

This study assessed the G6PD deficiency, SCT, and their co‐inheritance among 250 blood donors at the CCTH. Assessing the essential role G6PD plays in transfusion reactions and the implications of G6PD deficiency in transfusion reaction investigations brings to light the urgent need to determine its persistence in the population, associated relevant effects, and policy interventions for transfusion control. The findings demonstrate that a substantial proportion of otherwise healthy donors carried clinically relevant red cell abnormalities that are not routinely screened for during donor selection.

The demographic characteristics of the participants reveal that the majority were males, 146 (58.4%), had senior high school level education, 203 (81.2%), with the least having no educational background, 4 (1.6%). Most of the participants were single 221 (88.4%), settled in urban residence 126 (50.4%), and were students 184 (73.6%), with the least having a professional status 1 (0.4%). This donor population was primarily voluntary donors 185 (74.0%), most were first‐time donors 174 (69.6%), and the rest, who had donated before, at the time of sampling had donated once 35 (46.7%), with least of them having donated thrice 7 (9.3%), and majority of the donor participants donated at CCTH premises 64 (81.0%).

The majority of the study's donors were young and first‐time donors. Due to school‐based donation events and community mobilization initiatives, students and younger adults make up the majority of blood donors in Ghana and other sub‐Saharan African nations, as this demographic profile illustrates. Studies carried out in Berekum, Ghana [1], and Nigeria have shown similar donor demographics, with students and younger age groups making up the majority of the donor population [21]. This study found a comparatively higher percentage of female donors, in contrast to previous African studies that revealed a male predominance among donors. Because men are more likely to participate in blood donation campaigns and are less likely to be postponed owing to pregnancy, lactation, or anemia‐related conditions, previous research among blood donors in Ghana [1], Nigeria, and Saudi Arabia generally reported males as the dominating donor group [21, 25]. Therefore, shifting patterns of donor mobilization and increased female engagement in voluntary donation exercises within the study area may account for the comparatively higher female participation seen in this study. The large percentage of first‐time donors indicates the need for more effective donor retention tactics and better donor education, even while the preponderance of voluntary donors is positive from the standpoint of blood safety. Because they are more likely to undertake routine health screenings and have a better understanding of the standards for blood donation, repeat voluntary donors are typically linked to safer transfusion results [26, 27, 28].

The donors' unawareness of fundamental blood donation standards that might improve their health was another significant finding in this study. Add to this the extremely low level of donor knowledge about blood group, sickling status, G6PD status, and transfusion‐transmissible diseases (TTIs). Despite the relatively high prevalence of sickling and G6PD in Ghana and throughout West Africa, almost all participants were unaware of their status. However, awareness and knowledge of the demographic characteristics, coupled with laboratory investigation of their blood type, G6PD, and SCT status, play a central role in understanding the donor inclusion structure and thus provide a framework to update and revise the donor recruitment and inclusion policies. This finding raises important public health concerns because awareness of inherited hematologic disorders contributes significantly to preventive healthcare, reproductive counseling, and timely clinical management. Many people in countries without widespread newborn screening programs are unaware of their carrier or deficient status until they encounter issues or have unintentional testing. Gaps in donor education and health literacy among potential blood donors are further highlighted by the limited awareness of TTIs.

In the context of transfusion safety, the prevalence of G6PD deficiency found in this study is clinically significant. The reported incidence of 14% full deficiency suggests that about one in eight donor units may potentially come from a G6PD‐deficient donor, notwithstanding regional and ethnic variations in prevalence rates. This prevalence is comparable to results from other malaria‐endemic African settings and greater than those seen in a number of non‐African groups. A study by Liang et al. investigated G6PD deficiency screening and gene analysis in blood donors in Guangdong province and found an overall prevalence rate of G6PD deficiency, 6.97% [27]. Another study by Al‐alimi et al. identified an overall prevalence rate 11.5% of G6PD deficiency among the National University Students in the Middle Province (Ibb City), Yemen. Another corresponding study in Thailand among 514 blood donors identified a prevalence of 7.59% (35 [8.73%] males, 4 [3.54%] females) of G6PD deficiency blood [29]. Similar prevalence rates have been documented in research from Ghana, Nigeria, and other sub‐Saharan African countries, where partial protection against severe malaria has been associated with the persistence of G6PD deficiency. Adu et al. in Berekum [1], Ghana, found a prevalence rate similar to that of the current study, while Egesie et al. in Nigeria reported a 20.0% prevalence of G6PD deficiency among blood donors [21]. Studies from higher‐income nations and some non‐endemic areas, on the other hand, have shown significantly lower prevalence rates, which can be attributed to variations in population heterogeneity, genetic background, and malaria selection pressure [30, 31]. Thus, the very high prevalence found in this study is a reflection of the region's genetic epidemiology as well as the trait's historical selective advantage in malaria‐endemic people.

The results raise significant concerns about the transfusion of G6PD‐deficient blood to susceptible recipients from the standpoint of transfusion medicine. The antioxidant capacity and resistance to oxidative stress during storage or after transfusion may be compromised in red blood cells from G6PD‐deficient donors. Transfusion of G6PD‐deficient blood has been linked in a number of studies to increased hemolysis, elevated bilirubin levels, and decreased post‐transfusion red cell survival, especially in neonates, critically ill patients, and chronically transfused individuals like sickle cell disease or thalassemia patients [32, 33]. Due to their immature hepatic bilirubin metabolism, newborns are particularly susceptible to hyperbilirubinemia and kernicterus after being exposed to hemolyzed red blood cells [33].

SCT poses a threat as well in transfusion reactions or blood transfusion, assessing its current impacts in transfusion medicine, hence led to evaluating its co‐inherited prevalence with G6PD deficiency among blood donors as well. Of the 250 donors, 15 (6.0%) presented co‐inheritance of G6PD deficiency and SCT. Our findings correspond to a study conducted by Egesie et al., which recorded the prevalence of co‐infection of G6PD deficiency and SCT as 78 (5.4%) [21]. Another study by Alabdulaali et al. corroborates our study findings, recording 4 (0.35%) for both conditions.

Several studies on the prevalence of G6PD deficiency and co‐inheritance of G6PD‐deficient and SCT among blood donors reveal significant findings that influence researchers to recommend the inclusion of G6PD deficiency and SCT screening in the donation process. Our study findings affirm other research outcomes and recommendations, with our recorded prevalence of co‐inheritance of G6PD deficiency and SCT among blood donors at CCTH. The findings of our study revealed an overall significant prevalence of G6PD full deficiency and co‐inheritance of G6PD deficiency and SCT. Systematic reviews conducted in line with our study outcomes added that although the Hb levels of volunteer donors with G6PD deficiency trait, and SCT may be acceptable for blood donation, the influence of this regular blood donation on body iron storage and other iron metabolism parameters warrants further investigation, most importantly, studies in this area could be important for donor recruitment and retention [28]. Additionally, focusing on the various morphological and biochemical changes previously documented and understanding the molecular mechanisms related to these changes will be useful for developing preservation strategies to prevent deleterious effects during the processing and storage of blood products [28].

The study's hematological findings also shed light on donors' sex‐related physiological variations. The red blood cell count, hemoglobin concentration, hematocrit, and mean cell volume of male donors were significantly higher than those of female donors. This is in line with known physiological variations linked to androgenic stimulation of erythropoiesis and menstrual blood loss in females. On the other hand, platelet and white blood cell counts were noticeably greater among female donors. These results indicate the significance of routine hematological assessment in donor screening and blood safety review, even if they were not the study's main focus.

The findings of this investigation corroborate mounting data supporting focused screening guidelines for transfusion services in high‐prevalence environments. Selective screening techniques may increase transfusion safety, even though universal screening for G6PD deficiency and SCT may not currently be possible in many low‐resource nations due to budgetary and logistical limitations. Blood destined for infants, patients receiving chronic transfusions, recipients who are extremely ill, and exchange transfusion processes could all benefit from screening. Enhancing donor counseling and post‐donation education may also promote better donor practices and raise knowledge of inherited blood diseases.

This study emphasizes the necessity of more extensive public health education about inherited red cell diseases. Premarital counseling services, school health programs, child welfare clinics, and routine adolescent health assessments could all incorporate screening for sickle cell trait, G6PD deficiency, and blood group status to increase early awareness and lessen the burden of undiagnosed inherited hematologic conditions. Additionally, early detection may lessen the problems brought on by improper pharmaceutical exposure and promote informed reproductive choices.

This study has a number of limitations that should be noted despite the significant findings. First, the cross‐sectional design of the study, which was carried out at a single tertiary hospital, may restrict the findings' applicability to the larger Ghanaian donor population. Second, the methemoglobin reduction test employed for G6PD screening is a qualitative technique with low sensitivity for identifying intermediate‐deficient stages, especially in heterozygous females, despite being practical and affordable in environments with limited resources. As a result, some people with partial deficiencies might have been mistakenly labeled as normal. Similar to this, the sodium metabisulfite sickling test is mainly used for screening purposes and is unable to detect less frequent hemoglobin variations or differentiate between sickle cell trait and sickle cell illness. Even though confirmatory hemoglobin electrophoresis was performed, more advanced diagnostic techniques such as high‐performance liquid chromatography (HPLC) or molecular testing could provide greater diagnostic precision.

Additionally, storage lesions, oxidative stress indicators, post‐transfusion outcomes, and red cell survival in recipients transfused with G6PD‐deficient or SCT‐positive blood were not specifically evaluated in this investigation. Therefore, rather than using actual outcome data from this study population, the clinical implications highlighted are based on biologic plausibility and current literature. Larger sample volumes, quantitative G6PD testing, molecular characterization, and recipient outcome evaluations are thus necessary for future multicenter research.

## 5 Conclusion

The results of this study reveal that the overall prevalence of G6PD deficiency was recorded exclusively, although co‐inheritance of G6PD deficiency and SCT among blood donors was significant. This suggests that there is a necessity to screen blood donors for G6PD deficiency and SCT before donation. Several studies were found to be in line with our findings and ultimately our recommendations. Therefore, targeted public health interventions should be aimed at inclusion of G6PD deficiency and SCT screening in donation exercises, and further studies are recommended to research the effects of G6PD deficiency blood in transfusion outcomes.

## Author Contributions

**Bashirudeen Kofi Mensa Essel:** conceptualization, methodology, investigation, writing – original draft, project administration, data curation. **Walter Quarm:** conceptualization, methodology, investigation, writing – original draft, project administration, data curation. **Comfort Fynn:** investigation, methodology, writing – review and editing, conceptualization. **Rebecca Peniel Storph:** conceptualization, formal analysis, writing – original draft, methodology, investigation, data curation. **Evans Duah:** formal analysis, conceptualization, data curation, writing – review and editing, investigation, methodology. **Edward Morkporkpor Adela:** methodology, investigation, writing – review and editing, conceptualization. **Emmanuel Ofori:** writing – review and editing, methodology, conceptualization. **Nana Benyin Aidoo:** writing – review and editing, formal analysis, methodology, conceptualization. **Patrick Adu:** writing – review and editing, conceptualization, supervision, methodology.

## Funding

The authors have nothing to report.

## Conflicts of Interest

The authors declare no conflicts of interest.

## Data Availability

The data set for this study is available from the corresponding author and will be made available upon reasonable request.
